# Behavior underpins the predictive power of a trait‐based model of butterfly movement

**DOI:** 10.1002/ece3.5957

**Published:** 2020-03-06

**Authors:** Luke C. Evans, Richard M. Sibly, Pernille Thorbek, Ian Sims, Tom H. Oliver, Richard J. Walters

**Affiliations:** ^1^ School of Biological Sciences University of Reading Reading UK; ^2^ Syngenta Jealott's Hill International Research Centre Bracknell UK; ^3^ BASF SE, APD/EE Limburgerhof Germany; ^4^ Centre for Environmental and Climate Research University of Lund Lund Sweden

**Keywords:** body size, dispersal, Lepidoptera, motivation

## Abstract

1. Dispersal ability is key to species persistence in times of environmental change. Assessing a species' vulnerability and response to anthropogenic changes is often performed using one of two methods: correlative approaches that infer dispersal potential based on traits, such as wingspan or an index of mobility derived from expert opinion, or a mechanistic modeling approach that extrapolates displacement rates from empirical data on short‐term movements.
2. Here, we compare and evaluate the success of the correlative and mechanistic approaches using a mechanistic random‐walk model of butterfly movement that incorporates relationships between wingspan and sex‐specific movement behaviors.
3. The model was parameterized with new data collected on four species of butterfly in the south of England, and we observe how wingspan relates to *flight speeds*, *turning angles*, *flight durations*, and *displacement rates*.
4. We show that *flight speeds* and *turning angles* correlate with wingspan but that to achieve good prediction of displacement even over 10 min the model must also include details of sex‐ and species‐specific movement behaviors.
5. We discuss what factors are likely to differentially motivate the sexes and how these could be included in mechanistic models of dispersal to improve their use in ecological forecasting.

## Graphical abstract

Dispersal is often predicted using correlations with species traits. Here, we present new data that evaluate the success of the trait approach for predicting the movement behavior of four species of butterfly. We show that wingspan correlates well with flight speeds and turning angles but that to achieve good prediction of displacement over 10 min the model must also include the proportion of time spent flying.

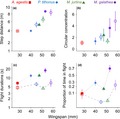

## 1 INTRODUCTION

Dispersal is a feature of animal behavior that crucially affects ecological processes across a range of spatial and temporal scales, including the persistence of species in fragmented landscapes, community dynamics, and ultimately evolutionary trajectories (Hanski, 1998; Nathan et al., 2008). To understand and predict the responses of species to anthropogenic pressures arising from changes in land‐use and climate change, it is necessary to understand both a species motivation and its capacity to move (Bonte et al., 2012; Gibbs, Saastamoinen, Coulon, & Stevens, 2010; Thompson & Gonzalez, 2017; Travis et al., 2013). These factors can be incorporated into process‐based mechanistic models (Doherty & Driscoll, 2018; Nathan et al., 2008). To date however mechanistic models have played only a limited, if important, role in ecological forecasting due to the time and costs required to acquire the necessary data for model parametrization and validation (Urban et al., 2016).

Given the challenges of developing these more complex mechanistic models, the pragmatic approach to applied questions concerning dispersal potential has often been to use a proxy of mobility, one that is either based on expert knowledge (Burke, Fitzsimmons, & Kerr, 2011; Shreeve, 1995) or simply correlated with another trait, such as body size (Bejan, 2000; Berwaerts, Van Dyck, & Aerts, 2002; Dudley & Srygley, 1994; Peters, 1986; Sekar, 2012). This approach, here termed the “correlative approach,” is valid at broad scales: search rates for foragers in two dimensions, for example, increase allometrically with body mass according to a power law with exponent 0.68 (Pawar, Dell, & Savage, 2012), and home range sizes increasing with exponent 1 for mammals (Jetz, Carbone, Fulford, & Brown, 2004). Natal dispersal distances increase with body mass in mammals and carnivorous bird species (Sutherland, Harestad, Price, & Lertzman, 2000), and similar relationships exist across a diverse range of taxa (Jenkins et al., 2007; Stevens et al., 2014), including freshwater fish (Shurin, Cottenie, & Hillebrand, 2009), marine fish (Bradbury, Laurel, Snelgrove, Bentzen, & Campana, 2008), and birds (Neuschulz, Brown, & Farwig, 2013; Paradis, Baillie, Sutherland, & Gregory, 1998). Relationships between traits and dispersal are however typically noisy and not uniform across guilds (Shurin et al., 2009; Sutherland et al., 2000). Consequently, accurate predictions of dispersal may require a nuanced understanding of the relationship between traits and the mechanisms influencing dispersal. This detail can typically only be provided for fewer species, a well‐known modeling trade‐off (Levins, (1966) still debated (Evans, Merow, Record, McMahon, & Enquist, 2016).

Butterflies present a useful system in which to compare the correlative and mechanistic approaches to forecasting dispersal rate. Mechanistic models have linked individual movement behavior to metapopulation dynamics (Ovaskainen & Hanski, 2004; Pe'er, Heinz, & Frank, 2006), home ranges size (Hovestadt & Nowicki, 2008; Kőrösi, Örvössy, Batáry, Kövér, & Peregovits, 2008), functional connectivity (Ovaskainen et al., 2008), minimum area requirements (Brown & Crone, 2016), and egg‐laying distributions (Evans, Sibly, et al., 2019; Grant, Parry, Zalucki, & Bradbury, 2018; Parry et al., 2017). Correlative approaches have linked levels of mobility to traits such as body size and wingspan (Kuussaari, Saarinen, Korpela, Pöyry, & Hyvönen, 2014; Sekar, 2012), and so these traits can partially explain interspecific variation in response to land‐use change (Öckinger et al., 2010). However, the correlative approach to forecasting dispersal rate remains contentious for several reasons. First, traits such as body size are only weakly associated with movement (Sekar, 2012); second, only rarely do we fully understand the reasons underlying interspecific variation (Stevens, Turlure, & Baguette, 2010); and third, large‐scale movement patterns are an emergent property of a complex interplay between movement capacity, individual behavior, and environmental influences (Nathan et al., 2008). A detailed understanding of how exactly body size impacts dispersal through the effects on observable small‐scale movement behavior is currently missing. Further, the extent to which mechanistic understanding can improve predictive power by comparison to a correlative approach is not well‐understood.

To inform comparison of correlative and mechanistic approaches, we here evaluate the effect of a trait often related to mobility, wingspan (Sekar, 2012), on measuring and forecasting movement in four species of butterfly in the south of England. We also present a random‐walk model of butterfly movement behavior that incorporates relationships between wingspan and the key aspects of the movement process, including flight speed, turning angle, and the proportion of time spent flying. We parameterize the model with newly collected data on butterfly flight paths. Though the number of species is limited, the collection of high‐precision movement and behavioral data allow us to closely evaluate the success of the two approaches and to explore why traits, such as body size or wingspan, may break down when predicting movement over longer timescales. The predictive success of the mechanistic approach is found to be strongly influenced by the inclusion of sex‐specific behavior, and we detail why the correlative approach is less successful.

## 2 METHODS

### 2.1 Study species and sites

The study was conducted on four species of grassland butterfly: *Aricia agestis* (Dennis & Schiffermüller, 1775)*, Maniola jurtina* (Linnaeus, 1758), *Pyronia tithonus* (Linnaeus, 1758), and *Melanargia galathea* (Linnaeus, 1758)*.* All four species are commonly found in southern England and range in average wingspan from around 28 to 58 mm (Thomas, 2010). The brown argus, *A. agestis,* is the smallest with a wingspan of between 25 and 31 mm (Newland, Still, Swash, & Tomlinson, 2015). It is a bivoltine species with adults found first on the wing in June and then again in August. In contrast, the other three species are univoltine, are found on the wing through June to September, and show marked sexual dimorphism in wingspan. Males are notably smaller than females on average in each case: the gatekeeper, *P. tithonus*: 37–43 versus 42–48 mm; the meadow brown, *M. jurtina*: 40–55 versus 42–60 mm; and the marbled white, *M. galathea*: ~53 mm versus ~58 mm (Newland et al., 2015). In this study, we take the mid‐value published values of wingspan as body size trait measure in further analyses. Sex was identified on the wing for all species except *A. agestis*, which required close inspection of caught individuals.

The study was conducted over two summers (2016: July–August and 2017: June–September) at three sites in the south of England: North Farm in Oxfordshire (51°37′N, 1°09′W), Jealott's Hill farm Berkshire (51°27′N, 0°44′W), and Sonning farm Berkshire (51°28′N, 0°53′W). The sites are representative of agricultural farms that have implemented agri‐environment schemes to promote biodiversity conservation. They consisted of a mixture of arable fields, open meadows, and nectar‐rich field margins. Habitats contained similar densities of flower resources and data from species were collected from across all sites to control for the effects of varying habitat composition on observed movement. The hourly air temperature was collected from meteorological stations deployed at two of the sites (Jealotts Hill & Sonning) and from the closest meteorological observation center for the third site North Farm (<3 km, RAF Benson).

### 2.2 Movement and behavioral observations

The behavior of individual butterflies was recorded in the field at a distance from observers of approximately 3 m for a maximum of 10 min between the hours of 10:00 and 16:00. During this observation period, the position of each individual was recorded by planting a sequentially numbered marker flag, either at each landing site or after every 15 s during continuous flight, following established methodology (Schultz, 1998; Turchin, 1991). To accurately record the movement of the butterflies, observations relied on two observers one placing the flags and the other constantly following and recording behavior. The precise location of each flag was retrospectively mapped using a high‐grade Global Navigation Satellite System receiver (Arrow 200 RTK). Observations were stopped early either if the butterfly could no longer be tracked (i.e., crossed hedges or lost from sight) or if a maximum number of 20 flags were used. Flight durations and behavior were recorded on a mobile phone using a bespoke Android App developed for the project (see Data availability statement).

Records of precise location, time, and behavior were later processed to calculate the distance between successive flags, hereafter referred to as a step distance. *Step speed* was calculated as *step distance*/*step duration*. *Step speed* was used as our measure of flight speed as step distances depend on both *step duration* and flight speed. *Turning angle* was calculated as the absolute subtended angle between successive steps (i.e., +40° and −40° were both recorded as 40°). *Flight duration* is calculated as the time between the observed takeoffs and landings of individual flights, thus a flight contains multiple step distances and turns. *Inter‐flight duration* was calculated as the time interval between successive flights. *Proportion of time flying* was calculated as the sum of flight time over the observation period. Finally, *displacement rate* is calculated as the Euclidean distance between positions at the start and the end of the observation period. *Aricia agestis* could only be sexed by catching the butterfly after the observation, reducing the number of observations in which sex was confirmed. Because of this, the sexes were pooled for comparison of step speeds, turns, and any displacement predictions in *A. agestis*.

### 2.3 Statistical analysis

Linear mixed effects models (LMERs) with a Gaussian error structure were used to evaluate the effect of wingspan, sex, and air temperature on the movement components. Butterfly ID was included in models of *flight duration, step speed,* and *turning angle* as random intercepts to account for repeated measures. Linear models were used for *proportion of time in flight* and *displacement rate* since in these analyses there was just one observation per individual. A single mean value of wingspan as reported in the literature was used for each sex x species combination and entered as a covariate in the analysis as a species/sex trait, alongside air temperature and sex as a fixed factor. Note that the focus of this study is to evaluate relationships to wingspan across groups and not the response to intraspecific variation in this particular trait. Model diagnostics were used to check the conformation of the data to the assumptions of the error structure, and suitable transformations were used when residuals were skewed. *Step speed* was square‐root transformed, *displacement rate* and *flight durations* were both log‐transformed, while *proportion of time flying* was logit‐transformed. To display the effects of circular concentration, a von Mises circular distribution was fitted to the turning angles and the parameter *k*, a reciprocal measure of the dispersion, was estimated with confidence intervals derived from a boot‐strapping procedure (Lund & Agostinelli, 2011). All analyses were conducted in R 3.6.1 (R Core Team, 2019). Mixed models were fitted using the “lme4” package (Bates, Mächler, Bolker, & Walker, 2015) with significance scores for coefficients estimated using the Satterthwaite method for approximating degrees of freedom through the “lmerTest” package (Kuznetsova, Brockhoff, & Christensen, 2017).

### 2.4 Mechanistic random‐walk model

A random‐walk model was designed around the empirical observations collected in this study in order to explore the effects of movement behaviors on predictions of displacement distances. The model runs at one second time intervals for a total of ten minutes of simulated time. An overview of the model is as follows: first, each individual draws from the observed distribution a proportion of time in flight, which multiplied by the total observation period duration, gives a total flight time during which the individual moves. To move during a flight, the individual draws from the observed distribution of step speeds and moves forward at that rate for 15 s. After 15 s, the individual changes heading by drawing a turning angle from a von Mises distribution fitted to the data. This process is repeated until the total flight time has elapsed. Distributions for the proportion of time in flight and step speeds were produced by interpolations on the empirical cumulative distribution functions fitted to the data. The model was built in NetLogo 6.0 (Wilensky, 1999), and analysis was carried out using the RNetLogo package (Thiele, 2014).

The effects of movement components on displacement rate and their relationship to wingspan were investigated in three scenarios differing in the distributions from which flight components were drawn. In the first scenario, *step speeds* were drawn from sex‐ and species‐specific distributions, but *turning angles* and *proportion of time in flight* were drawn from distributions of pooled data. In the second scenario, both *step speed* and *turning angles* were drawn from sex‐ and species‐specific distributions, while *proportion of time flying* was selected from a pooled distribution. In the third scenario, all distributions were sex‐ and species‐specific. For each scenario, the success of the model was evaluated by plotting the observed against the predicted mean displacement distances (Piñeiro, Perelman, Guerschman, & Paruelo, 2008). In each scenario, we conducted 50 repeats of the movement of 1,000 butterflies for each sex and species combination. For comparison with a trait‐based approach, we compare these results against the success of a simple regression of observed displacement against wingspan.

## 3 RESULTS

In total, detailed observations were undertaken on the flight paths and behavior of 583 individuals (*A. agestis:* 83; *M. jurtina*: ♀135, ♂155; *P. tithonus*: ♀75, ♂61; *M. galathea* ♀15, ♂58). Output from regressions evaluating the effects of wingspan, sex, and air temperature on the four components of movement and displacement rate are shown in Table 1. Movement was affected by the predictor variables in all cases except that sex had no effect on step speed or turning angle.

**Table 1:** Coefficients from LMERs and linear models (±standard errors) predicting the four components of the movement process and displacement rate from wingspan, sex, and air temperature

	Step speed (m/s) ±*SE*	Turning angle (°) ±*SE*	Flight duration (s) ±*SE*	Proportion of time flying ±*SE*	Displacement rate (m/s) ±*SE*
Wingspan (mm)	0.012*** ±0.001	−0.019** ±0.007	0.022*** ±0.006	0.040** ±0.015	0.037** ±0.01
Sex (M)	—	—	0.900*** ±0.077	1.931*** ±0.208	0.996*** ±0.036
Air temperature (°C)	—	−0.033* ±0.01	0.044*** ±0.011	0.186*** ±0.029	0.101*** ±0.02
Intercept	—	4.573*** ±0.43	−0.25 ±0.349	−8.684*** ±0.864	−8.256*** ±0.684
*N*	1,324	1,073	1,819	428	408
*R* ^2^	—	—	—	.257	.144
*R* ^2^ (c)	.33	.136	.42	—	—
*R* ^2^ (m)	.08	.025	.13	—	—

Note

Step speed was measured as distance moved in 15 s, turning angle is the change in heading between adjacent 15 s steps. Proportion of time flying and displacement rate were measured over 10 min. Non‐significant (*p* > .05) predictors are omitted from display.

* *p* < .05,

** *p* < .01,

*** *p* < .001.

**Figure 1:**
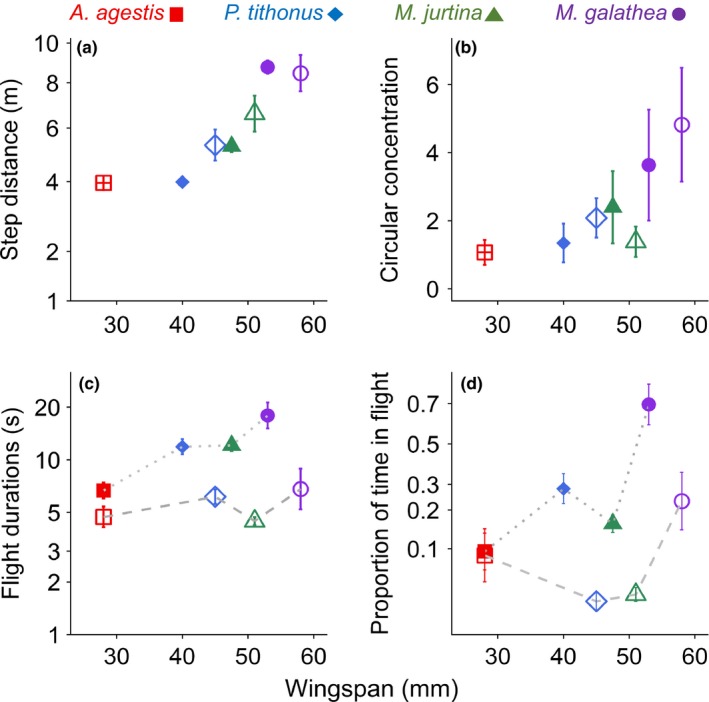
Effect of wingspan on (a) step distance over 15 s, equivalent to the step speed. (b) Von Mises circular concentration, *k,* of turning angle distribution. (c) Effect of wingspan and sex on log flight durations at air temperatures between 18 and 21°C; (d) logit transform of the proportion of time flying in 10 min observations. Females are shown as open symbols males as closed. Bars show standard errors. Dashed line connects females, and dotted line connects males

Step speed over 15 s was roughly proportional to wingspan (Table 1; Figure 1a), approximately doubling between the smallest butterflies, *A. agestis*, and the largest, *M. galathea*. Larger butterflies turned less between adjacent 15‐s steps, with the exception of female *M. jurtina* (Table 1; Figure 1b). Neither step speed nor turning angle were affected by sex, but turning angle reduced a little as air temperature increased.

Flight durations and proportion of time flying were affected by air temperature, sex, and wingspan (Table 1, Figure 1c,d). Butterflies flew longer and spent more time flying when it was warmer. The effects of sex and wingspan on flight durations are shown in Figure 1c for air temperatures between 18 and 21°C, the temperature window for which most data are available. Males flew much longer than females, more than three times longer in *M. jurtina*, and the largest species flew two to three times longer than the smallest. The effects of sex and wingspan on proportion of time flying are shown in Figure 1d. Males spent more time flying than females. There was variation between the sexes in the effects of wingspan but some tendency for larger species to spend more time flying.

**Figure 2:**
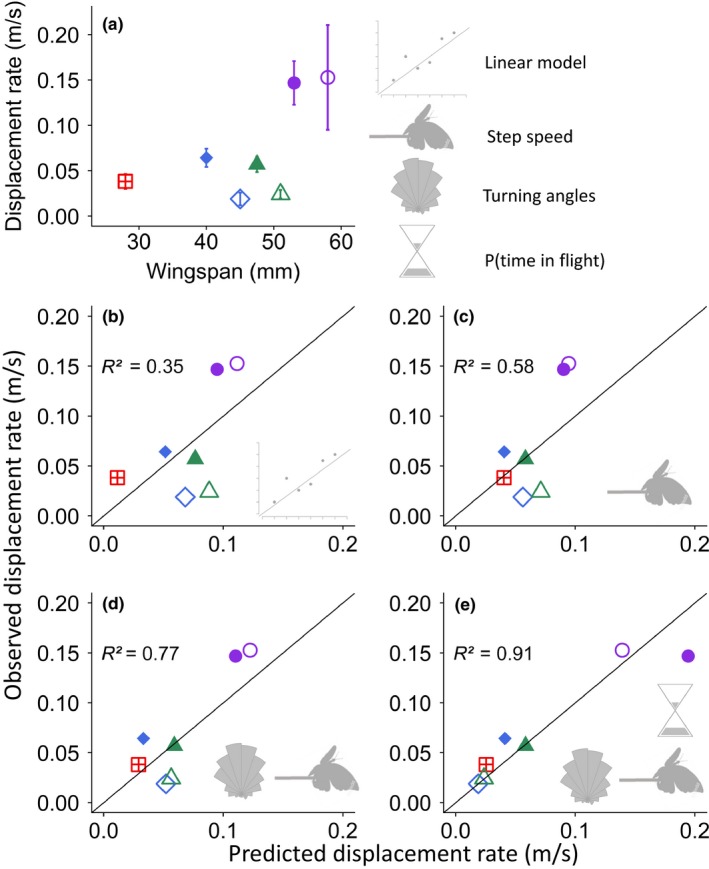
Observed and modeled mean displacement rates for each sex x species combination. Upper panel (a) shows relationship of displacement rates with wingspan as observed, bars show standard errors; lower panels show observed versus predicted displacement rates. Lines indicate perfect prediction. Symbols indicate modeling scenarios. (b) The correlative approach, fitting a regression line to the data in a; (c–e) mechanistic models. (c) Assuming only step speed is sex‐ and species‐specific; (d) assuming turning angle and step speed are sex‐ and species‐specific; (e) assuming all three movement components are sex‐ and species‐specific

Displacement rate is the total displacement observed during an observation bout (usually 10 min) divided by the bout duration and therefore gives the combined effects of all three movement components. Displacement rate was affected by air temperature, wingspan, and sex (Table 1), displacement rate was four times greater in the largest species, *M. galathea*, than in the smallest, *A. agestis* (Figure 2a), and the displacement rate of males was to 2–3 times that of females in *M. jurtina* and *P. tithonus.*

The success of the correlative approach, using a simple regression of displacement on wingspan, is shown in Figure 2b. The proportion of variance explained, *R* ^2^, is .35. For comparison, we developed a mechanistic model in which large‐scale movement patterns emerge from movement components (Section 2), with the aim of understanding how our measure of large‐scale movement, displacement rate, depends on step speed, turning angle, and the proportion of time flying. Using the model with only step speeds being sex and species‐specific, modeled displacement rate increases in proportion to wingspan, with a doubling between the smallest species *A. agestis* and the largest butterfly, females of *M. galathea*, but this is less than that observed (Figure 2c), and this gives an *R* ^2^ of .58. When the effects of turning angle are also made sex‐ and species‐specific the fit improves with *R* ^2^ rising to .77 (Figure 2d). The most realistic model makes all three movement components sex‐ and species‐specific and gives an *R* ^2^ of .91 (Figure 2e). Modeled displacement rate then increases fourfold with wingspan between the smallest and the largest butterflies, and females of *P. tithonus* and *M. jurtina* have a lower displacement rate than *A. agestis* (Figure 2e), very like the pattern of observed displacement rates (Figure 2a), and much better than the results of the correlative approach (Figure 2b).

## 4 DISCUSSION

The full mechanistic model, which included sex‐specific movement behaviors, outperformed simpler models containing only the effects of changing step speeds and turning angles and dramatically outperformed the correlative approach in predicting displacement rate, as shown by comparing Figure 2b,e. In building the mechanistic model, we took into account that the distance moved by an individual is a combination of movement capacity, behavior, and environmental influence (Nathan et al., 2008), so we began by looking at each of these components to understand how a widely used species trait for approximating movement, wingspan, relates to small‐scale individual movement. We then explored the subsequent effects of these including these components in mechanistic models predicting longer‐term displacement. Movement capacity was strongly related to wingspan. Larger butterflies had a greater capacity for movement than smaller butterflies as they flew faster and straighter irrespective of sex (Figure 1). Behavior was also related to wingspan with larger butterflies found to have longer flights than smaller butterflies, though sex (Figure 1c) and air temperature were also important (Table 1). Finally, the proportion of time in flight, which is a combination of flight and inter‐flight durations, increased with wingspan, though the most important factors were sex (Figure 1d) and air temperature (Table 1). The proportion of time in flight, which measures variation in behavior, therefore decoupled the linear relationship between wingspan and displacement rate. By comparison with the correlative approach (*R* ^2^ = .35) including sex‐ and species‐specific variations in the proportion of time in flight produced substantially more accurate medium‐term displacement predictions (*R* ^2^ = .91).

The relationship between wingspan and flight speed (Figure 1) is expected from first‐principle scaling arguments assuming isometry with changing size (Norberg & Rayner, 1987). Previous intraspecific comparisons based on temperate species have found flight speed correlates well to wingspan (Berwaerts et al., 2002), but interspecific comparisons conducted on a large sample of neotropical species, where isometry is likely violated, have found mixed results (Dudley, 1990; Dudley & Srygley, 1994, 2008). Wingspan alone provided a good predictor of flight speed in our study based on four related species living in a shared habitat; however, extrapolation of these results to multiple species will also need to account more directly for traits such as wing loading, aspect ratio, and momentum of inertia arising from differences in wing shape and proportions (Betts & Wootton, 1988).

The relationship between wingspan and directedness of flights for butterflies has not received much attention even though turning angles are commonly reported. We found that the larger butterflies had straighter flights (Figure 1b) and that inclusion of species/sex‐specific turning angles improved displacement predictions considerably (Figure 2d). Size might influence directedness if the higher inertia of larger butterflies leads to decreases in maneuverability producing fewer or shallower turns as is the case for bats (Norberg & Rayner, 1987). Detailed studies of butterfly movement in real‐time conducted using harmonic radar suggest turning angle also varies within a species among habitats (Cant, Smith, Reynolds, & Osborne, 2005), likely reflecting a change in foraging strategy in response to resource density and/or landscape features (Delattre et al., 2010; Fownes & Roland, 2002; Odendaal, Turchin, & Stermitz, 1989; Roland, Keyghobadi, & Fownes, 2000; Schtickzelle, Joiris, Dyck, & Baguette, 2007; Zalucki & Kitching, 1982). It remains to be determined to what extent turning angle varies between species and how it subsequently influences displacement rates. Further, it is not well explored how variation in turning relates to sex‐ and species‐specific search strategies (Root & Kareiva, 1984) for the location of different resources.

Larger butterflies flew for longer than smaller butterflies though there was a strong effect of both sex and temperature. The influences of size and temperature on flight durations are consistent with previous studies (Cormont et al., 2011; Heinrich, 1986) and theoretical predictions based on the physics, anatomy, and posture of *Colias* species (Kingsolver, 1983; Tsuji, Kingsolver, & Watt, 1986). However, the substantial behavioral differences between the sexes demonstrate the limitations of using a single trait, such as wingspan, to predict mobility. Male flight behavior may primarily reflect a search for females and repeat matings, a behavior termed “patrolling” (Brakefield, 1982; Shreeve, 1984), whereas females are primarily focused on locating suitable egg‐laying sites and avoiding the unwanted attentions of males. Flight durations and inter‐flight periods are also likely subject to the spatial distribution of nectar resources and egg‐laying sites (Odendaal et al., 1989; Root & Kareiva, 1984), which suggests the extent of differences among the sexes and species may also be dependent on the resources in the immediate environment. These factors altogether likely explain why in practice traits such as wingspan are only weak predictors of mobility. However, by accounting for these differences we demonstrate that it is possible to predict displacement rates with a higher degree of accuracy (Figure 2).

While it is clear that wingspan provides an important trait‐based approach to modeling dispersal potential, the accuracy of model predictions was contingent on sex‐ and species‐specific trait parameterizations (Figure 2e), particularly for flight durations. For instance, though females flew slightly faster and straighter than males of the same species (Figure 1), they spent substantially less time in flight, leading to much lower overall displacement rates (Figure 2a). These differences ultimately influenced interspecific comparisons, with females of *P. tithonus* and *M. jurtina* showing lower displacement rates than the slower and more tortuous flying *A. agestis*. The influence of behavior is, therefore, a key factor in explaining variation in movement (Morales & Ellner, 2002) which is less directly related to morphological traits such as wingspan. Further, though our study measured local movements within homogenous habitat, the discrepancy between trait and dispersal is likely further uncoupled across different quality habitat patches which have been shown to have strong effects on butterfly behavior (Conradt, Bodsworth, Roper, & Thomas, 2000; Delattre et al., 2010; Odendaal et al., 1989; Roland et al., 2000; Schtickzelle et al., 2007). This is particularly relevant for understanding the role of rarer long distances movements in connecting populations which, though likely influenced by a capacity for movement, may be crucially influenced by behavior and interactions with the landscape structure (Nowicki et al., 2014). This context‐dependency demonstrates a common weakness of the mechanistic approach, as it necessitates the collection of detailed behavioral information of the target species across different many circumstances. A useful contribution of mechanistic models of movement is therefore in explaining the basis of motivational differences in relation to resource density, habitat structure, and foraging strategies, such that they provide better prediction for dispersal for species across varying landscape structures (Doherty & Driscoll, 2018; Johnston et al., 2019; Patterson, Thomas, Wilcox, Ovaskainen, & Matthiopoulos, 2008; Pauli et al., 2013; Urban et al., 2016).

In conclusion, we have shown that the reason wingspan can serve as a proxy for dispersal in butterflies is that it correlates well with flight speed and the tortuosity of butterfly movement. However, the most accurate predictions of displacement depend on sex‐ and species‐specific parameterizations of flight and inter‐flight durations, which decouple the relationship between wingspan and movement rate. Since these behaviors likely reflect motivation to move, substantive improvements in model predictions will require an understanding of how species view and utilize resource availability in complex landscapes. Demonstrating the extent to which behavior can improve predictive power over simple correlative approaches suggests that there is both scope and strong justification to develop process‐based models as a practical tool for ecological forecasting.

## CONFLICT OF INTEREST

None declared.

## AUTHOR CONTRIBUTIONS

LE collected the data, conducted the analysis, and developed code for the individual‐based model. LE, RS, and RW led the writing of the manuscript with contributions from TO, IS, and PT.

## Data Availability

Data from the study and the app used for the project are archived at Mendeley Data: http://dx.doi.org/10.17632/kpcgkfmpv8.1. Full data description is provided by Evans, Sims, et al. (2019).
